# Head kinematics in patients with neck pain compared to asymptomatic controls: a systematic review

**DOI:** 10.1186/s12891-022-05097-z

**Published:** 2022-02-16

**Authors:** Esther Franov, Matthias Straub, Christoph M. Bauer, Markus J. Ernst

**Affiliations:** Zurich University of Applied Sciences, School of Health Professions, Institute of Physiotherapy, Katharina-Sulzer-Platz 9, 8400 Winterthur, Switzerland

**Keywords:** Neck pain, Biomechanical phenomena, Head movements, Movement tasks, Whiplash

## Abstract

**Background:**

Neck pain is one of the most common musculoskeletal disorders encountered by healthcare providers. A precise assessment of functional deficits, including sensorimotor control impairment, is regarded necessary for tailored exercise programmes. Sensorimotor control can be measured by kinematic characteristics, such as velocity, acceleration, smoothness, and temporal measures, or by assessing movement accuracy. This systematic review aims to identify movement tasks and distinct outcome variables used to measure kinematics and movement accuracy in patients with neck pain and present their results in comparison to asymptomatic controls.

**Methods:**

Electronic searches were conducted in MEDLINE, PEDro, Cochrane Library and CINAHL databases from inception to August 2020. Risk of bias of included studies was assessed. Movement tasks and specific outcome parameters used were collated. The level of evidence for potential group differences in each outcome variable between patients with neck pain and controls was evaluated.

**Results:**

Twenty-seven studies examining head kinematics and movement accuracy during head-aiming, functional and unconstrained movement tasks of the head were included. Average Risk of Bias of included studies was moderate. In total, 23 different outcome variables were assessed. A strong level of evidence for an increased *movement time* and for an increased *number of errors* during head aiming tasks was found. Moderate evidence was found in traumatic neck pain for a decreased *mean velocity*, *peak acceleration*, and *reaction time*, and for *point deviation* and *time on target* during head aiming tasks. Moderate evidence was found for decreased *acceleration* during unconstrained movements, too.

Results on the remaining movement task and outcome variables showed only limited, very limited or even conflicting level of evidence for patients with neck pain to differ from controls.

**Conclusions:**

Sensorimotor control in NP in the way of kinematic and movement accuracy characteristics of head motion was examined in head aiming, functional or unconstrained movement tasks.

The results from this review indicate that for some characteristics that describe sensorimotor control, patients with NP differ from healthy controls.

**Systematic review registration:**

PROSPERO registration number: CRD42020139083.

**Supplementary Information:**

The online version contains supplementary material available at 10.1186/s12891-022-05097-z.

## Background

Neck pain (NP) is a worldwide common and often recurrent disorder [1], with a 1-year prevalence of 39% and a point prevalence of 13% in the adult population [2]. NP can lead to disability [3] and generate high health care and economic costs, due to work absenteeism and presenteeism [4]. After low back pain, NP is one of the most common musculoskeletal disorders encountered by health professionals [5]. Current clinical guidelines recommend active rehabilitation, including exercises, to restore optimal function [6]. Tailored exercise programmes that address individual functional deficits are regarded superior to general physical activity [7] or general neck exercises [8] in reducing NP and disability. Therefore, a precise assessment of functional deficits in patients with NP should form the base for any individually-targeted active treatment approach [9].

Functional impairments frequently seen in patients with NP are a reduced range of motion [10], neuromuscular disturbances [11] and alterations in sensorimotor control [12].

Reimann and Lephard [13], described the sensorimotor control system, “incorporates all the afferent, efferent, and central integration and processing components involved in maintaining joint stability”. Afferent information is given by the visual and vestibular systems, as well as the peripheral mechanoreceptors (e.g., muscle spindles). In patients with NP, cervical proprioceptive input can be altered by pain, direct damage to joints or muscles, functional impairments or morphological changes in neck muscles, and can consequently lead to impaired sensorimotor control [9]. Functional alterations that indicate impairment of the cervical sensorimotor control system in patients with NP have been reported for eye movement control [14], postural stability [15], eye-head [16] and head-trunk coordination [17], joint position- [18], force- [19] and movement sense [20]. Impaired sensorimotor control can be observed in the movement itself by measuring and quantifying its kinematics [21]. Kinematics describe the motion of objects in space (such as the head) and the most common method is to study their position vector. This aspect has been examined widely in NP studies and is frequently described as range of motion, as well as other position vector-related measures [10, 21]. Less well studied are the time derivatives of the position vector, such as velocity (1st derivative), acceleration (2nd derivative) or jerk (3rd derivative), which can give further insight into the quality of the movement. Another movement related aspect is the aforementioned movement sense, which can be assessed by the ability to precisely follow a given path with a head mounted point projection, further described here as movement accuracy.

Two recent reviews on kinematics of head movements in patients with NP compared to a control group reported either on velocity [10] or on functional tasks [22], however, there has been no review examining further kinematic quantities such as acceleration, jerk or other time-domain related parameters, and for different kinds of movement tasks.

Further reviews on the topic of sensorimotor control in NP focussed primarily on position sense, but give an only incomplete overview regarding movement accuracy [23, 24]. So far, no review has examined which of various variables describing kinematics and movement accuracy might be best suited for distinguishing patients with NP from asymptomatic controls.

Accordingly, this systematic review aims to give an overview of movement tasks and outcome measures used to examine head kinematics and movement accuracy in patients with NP compared to asymptomatic controls through critical appraisal of the published literature. An additional aim is to examine the evidence for their ability to discriminate between individuals with and without NP.

## Methods

### Review registration

The protocol for this review was registered with the International Prospective Register of Systematic Review (PROSPERO) in April 2020 (CRD42020139083). The review process was conducted using the guidelines of The Preferred Reporting Items for Systematic Reviews and Meta- Analyses (PRISMA) [25].

### Publications and participants

Studies on adults of both sexes with acute, subacute, or chronic idiopathic and traumatic (whiplash associated disorder = WAD) NP were included in the review. Studies with focus on specific NP conditions, e.g., radiculopathy, myelopathy, or post-surgical studies, were excluded. Studies could be of cross-sectional or longitudinal nature but had to incorporate a healthy control group.

Only full text studies published in English were included. No limitation on publication date was applied.

### Outcomes

Outcome measures had to be reported as parameters of head motion kinematics or movement accuracy for the NP and the control group.

### Search strategy and study selection

Electronic searches were conducted in the databases MEDLINE (via Ovid SP), PEDro, Cochrane Library and CINAHL (via ebscohost.com) from inception until August 2020.

MeSH terms that described the NP conditions, biomechanical phenomena, measurement properties and study design were selected by two reviewers (EF, MS). The complete search strategy used for MEDLINE is reported in Additional file 1.

Supplemental, online platforms of large publishers, including ScienceDirect, Informa Healthcare, SpringerLink and Wiley Online Library, were searched using well-known authors in the field. Reference lists of included studies were hand-searched, and Web of Science was checked for citations of included studies. Eligible citing and referenced studies were included until September 2020.

Studies identified in the search were downloaded into EndNote X9 (Clarivate Analytics, USA) and duplicates were removed. Study selection was conducted independently by two reviewers (EF and MS) and interrater agreement was calculated using Cohen’s kappa. Raters discussed any discrepancies and as required, consulted a third author (MJE) until consensus was reached. Identified publications were screened primarily by title and abstract. The selected studies were further assessed for eligibility by full text reading and the reasons for study exclusion were documented (see Table 1 at the end).

**Table 1:** List of excluded studies with reason

Study	Reason for exclusion
Alsultan F, Cescon C, De Nunzio AM, Barbero M, Heneghan NR, Rushton A, et al. Variability of the helical axis during active cervical movements in people with chronic neck pain. Clinical biomechanics (Bristol, Avon). 2019;62:50–7 [26].	No outcome of interest
Bahat HS, Croft K, Hoddinott A, Carter C, Treleaven J. Remote kinematic e-training for patients with chronic neck pain, a randomised controlled trial. Manual Therapy. 2016;25:e35 [27]	Conference abstract
Bahat HS, Sprecher E, Sela I, Treleaven J. Neck motion kinematics: an inter-tester reliability study using an interactive neck VR assessment in asymptomatic individuals. European Spine Journal. 2016;25 (7):2139–48 [28].	No control group
de Zoete RMJ, Osmotherly PG, Rivett DA, Snodgrass SJ. Cervical Sensorimotor Control Does Not Change Over Time and Is Not Related to Chronic Idiopathic Neck Pain Characteristics: A 6-Month Longitudinal Observational Study. Physical therapy. 2020;100 (2):268–82 [29]	Sample duplicate
Geisinger D, Ferreira E, Suarez A, Suarez H. Dynamic modeling and experimental results for a head tilt response. Conference Proceedings: Annual International Conference of the IEEE Engineering in Medicine & Biology Society. 2010;2010:2986–9 [30].	No outcome of interest
Goncalves C, Silva AG. Reliability, measurement error and construct validity of four proprioceptive tests in patients with chronic idiopathic neck pain. Musculoskeletal science & practice. 2019;43:103–9 [31]	No outcome of interest
Grip H, Jull G, Treleaven J. Head eye co-ordination using simultaneous measurement of eye in head and head in space movements: potential for use in subjects with a whiplash injury. J Clin Monit Comput. 2009;23:31–40 [32].	Missing data
Jull G, Amiri M, Bullock-Saxton J, Darnell R, Lander C. Cervical musculoskeletal impairment in frequent intermittent headache. Part 1: Subjects with single headaches. Cephalalgia. 2007;27 (7):793–802 [33].	No outcome of interest
Kristjansson E, Dall’alba P, Jull G. Cervicocephalic kinaesthesia: reliability of a new test approach. Physiotherapy Research International. 2001;6 (4):224–35 [34].	No control group
Kristjansson E, Björnsdottir SV, Oddsdottir GL. The long-term course of deficient cervical kinaesthesia following a whiplash injury has a tendency to seek a physiological homeostasis. A prospective study. Man Ther. 2016;22:196–201 [35]	No control group
Lascurain-Aguirrebena I, Newham DJ, Galarraga-Gallastegui B, Critchley DJ. Differences in neck surface electromyography, kinematics and pain occurrence during physiological neck movements between neck pain and asymptomatic participants. A cross-sectional study. Clinical biomechanics (Bristol, Avon). 2018;57:1–9 [36]	No outcome of interst
Meisingset I, Stensdotter AK, Woodhouse A, Vasseljen O. Changes in neck motion and motor control and associations with neck pain in patients with non-specific neck pain. Physiotherapy. 2015;101:e994 [37]	Conference abstract
Meisingset I, Stensdotter AK, Woodhouse A, Vasseljen O. Neck motion, motor control, pain and disability: A longitudinal study of associations in neck pain patients in physiotherapy treatment. Manual Therapy. 2016;22:94–100 [38]	No control group
Oddsdottir GL, Kristjansson E. Two different courses of impaired cervical kinaesthesia following a whiplash injury. A one-year prospective study. Man Ther. 2012;17 (1):60–5 [39]	No control group
Roijezon U, Bjorklund M, Bergenheim M, Djupsjobacka M. A novel method for neck coordination exercise--a pilot study on persons with chronic non-specific neck pain. J Neuroeng Rehabil. 2008;5:36 [40]	No control group
Rudolfsson T, Djupsjobacka M, Hager C, Bjorklund M. Effects of neck coordination exercise on sensorimotor function in chronic neck pain: a randomized controlled trial. Journal of Rehabilitation Medicine 2014 Oct;46 (9):908–914. 2014 [41]	No control group
Saadat M, Salehi R, Negahban H, Shaterzadeh MJ, Mehravar M, Hessam M. Traditional physical therapy exercises combined with sensorimotor training: the effects on clinical outcomes for chronic neck pain in a double-blind, randomized controlled trial. Journal of Bodywork and Movement Therapies 2019 Oct;23 (4):901–907. 2019 [42]	No control group
Sarig Bahat H, Weiss PL, Sprecher E, Krasovsky A, Laufer Y. Do neck kinematics correlate with pain intensity, neck disability or with fear of motion? Journal of the Israeli Physical Therapy Society (JIPTS). 2014;16 (2):38- [43]	No control group
Sarig Bahat H, Takasaki H, Chen X, Bet-Or Y, Treleaven J. Cervical kinematic training with and without interactive VR training for chronic neck pain – a randomized clinical trial. Manual Therapy. 2015;20 (1):68–78 [20]	No control group
Bahat HS, Croft K, Carter C, Hoddinott A, Sprecher E, Treleaven J. Remote kinematic training for patients with chronic neck pain: a randomised controlled trial. European Spine Journal. 2018;27 (6):1309–23 [44]	No control group
Treleaven J, Croft K, Carter C, Hoddinott A, Sarig-Bahat H. Are functional complaints relating to neck motion related to altered cervical kinematics in those with neck pain? Musculoskeletal Science and Practice. 2017;28:e12 [45]	Conference abstract
Treleaven J, Chen X, Sarig Bahat H. Factors associated with cervical kinematic impairments in patients with neck pain. Manual Therapy. 2016;22:109–15 [46]	No control group
Treleaven J, Takasaki H, Grip H. Altered trunk head co-ordination in those with persistent neck pain. Musculoskeletal Science and Practice. 2019;39:45–50 [17]	No outcome of interest
Tsang SM, Szeto GP, Lee RY. Relationship between neck acceleration and muscle activation in people with chronic neck pain: Implications for functional disability. Clinical Biomechanics. 2016;35:27–36 [47]	Sample duplicate
Waeyaert P, Jansen D, Bastiaansen M, Scafoglieri A, Buyl R, Schmitt M, et al. Three-dimensional Cervical Movement Characteristics in Healthy Subjects and Subgroups of Chronic Neck Pain Patients Based on Their Pain Location. Spine. 2016;41 (15):E908–14 [48]	Missing data
Werner IM, Ernst MJ, Treleaven J, Crawford RJ. Intra and interrater reliability and clinical feasibility of a simple measure of cervical movement sense in patients with neck pain. BMC Musculoskeletal Disorders. 2018;19 (1):358 [49]	No control group
Williams G, Sarig-Bahat H, Williams K, Tyrrell R, Treleaven J. Cervical kinematics in patients with vestibular pathology vs. patients with neck pain: A pilot study. Journal of Vestibular Research. 2017;27 (2–3):137–45 [50]	Only neck patients with reduced mean velocity were included
Woltring HJ, Long K, Osterbauer PJ, Fuhr AW. Instantaneous helical axis estimation from 3-D video data in neck kinematics for whiplash diagnostics. Journal of Biomechanics. 1994;27 (12):1415–32 [51]	No outcome of interest
Zito G, Jull G, Story I. Clinical tests of musculoskeletal dysfunction in the diagnosis of cervicogenic headache. Manual Therapy. 2006;11 (2):118–29 [52].	No outcome of interest

### Assessment of methodological quality

Assessment of the methodological quality of included studies was conducted using an adapted form of the Quality Assessment Tool for Observational Cohort and Cross-Sectional Studies of the U.S. National Heart, Lung, and Blood Institute [53]. This tool has been used in similar systematic reviews to assess a potential risk of bias (RoB) [22, 24]. After pilot testing, the original form was tailored to fit the case control design of included studies and the review’s aims. In total fourteen items remained.

All items were compared by both reviewers for their weight in assessing risk of bias (RoB). It was decided to double the value of six items addressing the RoB more exclusively (items 5,6,8–10, 13), resulting in a maximum score of twenty points. For the total methodological quality rating (out of a maximum score of 20 points), a score of > 13 was interpreted as the study having a low RoB; a score from 7 to 13 a moderate RoB; and a score < 7 a high RoB.

A detailed description of the implemented adaptations is presented in Additional file 2.

Quality assessment was performed by two independent reviewers (EF and MS). A third independent reviewer (MJE) was consulted in case of disagreement.

### Data extraction and synthesis

A data extraction form was developed. Two reviewers independently completed the form for each study and cross-checked the extracted data. Data included information on sampling procedures, sample sizes, participant data, measurement methods, outcome variables and RoB. If results were presented in figures only, WebPlotDigitizer would have been used to extract numerical data [54]. This semi-automatic extraction tool has previously been proven reliable and valid [55]. Differences between groups were considered statistically significant, if a hypothesis testing revealed a *p*-value < 0.05.

Included studies were assessed for methodological homogeneity, considering their potential for meta-analysis.

Findings were summarized for any outcome variable and across studies and labelled with a corresponding “Level of evidence”, according to an adapted classification system, mentioned by the method guidelines for systematic reviews in the Cochrane collaboration Back Review Group [56] (Table 2). The number and methodological quality of studies, and the consistency of results between studies, were considered.

**Table 2:** Level of evidence

Level of evidence	Criteria
Strong	Multiple studies with low RoB AND consistent findings across all studies
Moderate	One study with low RoB AND/OR multiple studies of moderate RoB AND consistent findings across all studies
Limited	One study with moderate RoB AND consistent findings across all studies
Very limited	One study with high RoB
Conflicting	Inconsistent findings between studies

*RoB* Risk of Bias

The summary of findings and level of evidence for kinematic and movement accuracy measures are presented separately for idiopathic NP (INP) and WAD, each group in comparison to healthy controls. For outcome assessed by studies without differentiation of NP onset (in future described as “unclassified NP”, the summary of findings and level of evidence was evaluated together with the results of INP studies.

Consistent findings were defined a priori as differences of NP subgroups compared to healthy controls indicating in the same direction.

## Results

### Literature search results

Database and hand-searching identified 1′000 publications. After removal of duplicates, 870 records remained. These were further screened by title and abstract reading, leading to 814 studies being excluded, with an interrater agreement for exclusion of .85. No aspects of either kinematics or movement accuracy were examined and/or participants suffered from specific NP conditions were the main reasons for exclusion. Subsequently, 56 studies were selected for full text reading. Finally, 27 studies were included in the review with the full agreement of both reviewers (see Table 1: List of excluded studies).

**Fig. 1:**
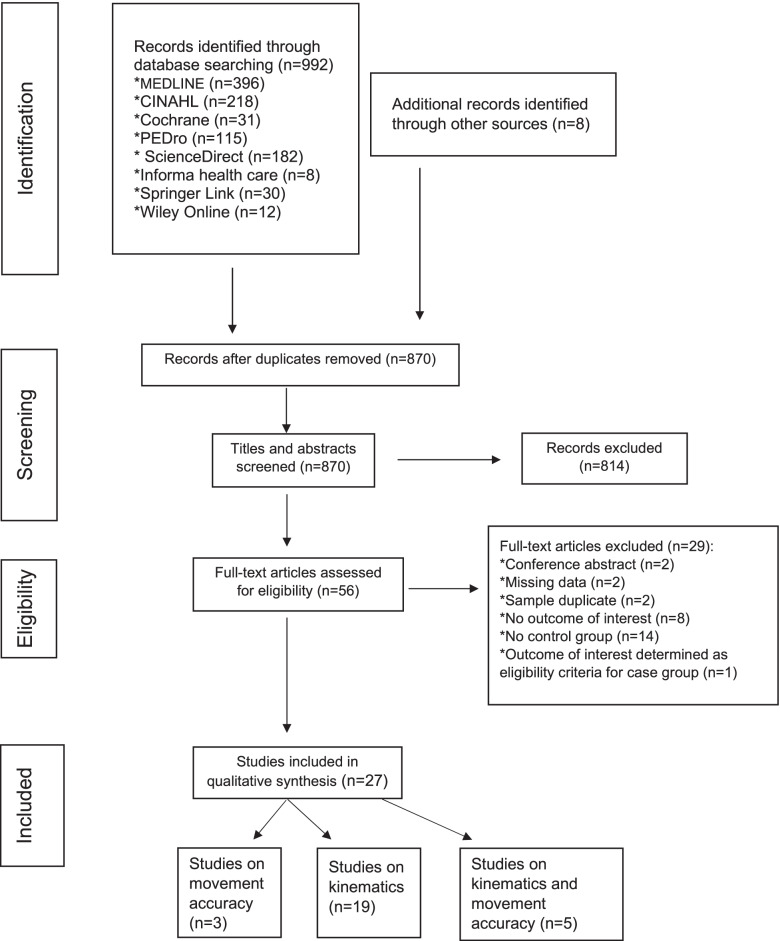
Flow chart of study selection process

Figure 1 illustrates the flow of studies through the selection process.

### Methodological quality of included studies

The overall rating of the methodological quality of included studies ranged from 4 to 15 (out of 20 points), with an average score across studies of 9.5, indicating an overall moderate RoB. Cohen’s kappa for interrater reliability, and before consensus discussion, was .81. Disagreements between the two reviewers could be solved by discussion (See Table 3 for details on critical appraisal). All studies stated a clear review question, and only three [61, 63, 79] did not provide appropriate descriptive data. There was a lack of information to prevent selection bias in all studies, due to missing information on the sampling process, population characteristics or participation rate. Only three studies provided a sample size calculation [57, 59, 75]. Two studies gave no description of the definition for the control group [71, 83]. Eleven studies had missing information on the reliability of outcome measurements [57, 58, 61, 63, 67, 72–74, 77, 82, 83]. Confounding occurred, either by the application of different exclusion criteria across groups, by not having controlled for factors such as age, sex or comorbidities during the selection process. Another source of confounding was an insufficient control of further movement task specifications, such as speed, or displacement. Only five studies gave sufficient information for these RoB items [60, 75, 76, 78, 79]. One study [70] mentioned that the statistician was not blinded, none of the other studies reported on blinding of outcome assessors or statisticians.

**Table 3:** Methodological quality assessment of included studies

Publication	Research question or objective clearly stated	Selection						Method			Statistics				Score	RoB
		Population clearly specified and defined	Participation rate of eligible persons at least 50%	All subjects recruited from similar population	Eligibility criteria pre-specified^**a**^	Potentially confounding co-morbidities excluded	Sample size justification provided	Exposure measures clearly defined, valid, reliable ^**a**^	Outcome measure clearly defined, valid, reliable ^**a**^	Exposure measured at day of outcome assessment	Different levels of exposure examined	Statistician blinded to exposure status of participants	Key potential confounding variables adjusted statistically	Appropriate descriptive statistics presented	Total	Overall risk
Baydal-Bertomeu et al. [57]	✓	X	NR	X	**X**	**X**	✓	**✓**	**X**	**NR**	X	NR	**✓**	✓	7	moderate
Descarreaux et al. [58]	✓	X	NR	X	**X**	**X**	X	**✓**	**X**	**X**	X	NR	**X**	✓	4	moderate
De Zoete et al. [59]	✓	✓	NR	✓	**✓**	**X**	✓	**✓**	**✓**	**x**	✓	NR	**✓**	✓	14	low
Ernst et al. [60]	✓	✓	NR	X	**✓**	**✓**	X	**✓**	**✓**	**✓**	X	NR	**✓**	✓	15	low
Gadotti et al. [61]	✓	X	NR	x	✓	✓	x	✓	X	**NR**	x	NR	X	X	7	moderate
Grip et al. [62]	✓	X	NR	X	**✓**	**X**	X	**✓**	**✓**	**✓**	✓	NR	**X**	✓	11	moderate
Hage et al. [63]	✓	x	NR	x	✓	**x**	x	✓	**X**	**NR**	X	NR	**X**	X	5	high
Kristjansson et al. [64]	✓	X	NR	X	**✓**	**X**	x	**✓**	**✓**	**NR**	X	NR	**X**	✓	8	moderate
Kristjansson & Oddsdottir [65]	✓	X	NR	X	**✓**	**X**	X	**✓**	**✓**	**X**	✓	NR	**✓**	✓	11	moderate
Lemmers et al. [66]	✓	X	NR	X	**✓**	**X**	x	**✓**	**✓**	**NR**	X	NR	**✓**	✓	10	moderate
Meisingset et al. [67]	✓	X	NR	X	**✓**	**X**	X	**✓**	**X**	**✓**	✓	NR	**✓**	✓	11	moderate
Oddsdottir et al. [68]	✓	X	NR	X	**✓**	**X**	X	**✓**	**✓**	**X**	✓	NR	**✓**	✓	11	moderate
Ohberg et al. [69]	✓	X	NR	X	**✓**	**X**	X	**✓**	**✓**	**NR**	X	NR	**X**	✓	8	moderate
Osterbauer et al. [70]	✓	X	NR	X	**X**	**X**	X	**✓**	**✓**	**NR**	X	X	**X**	✓	6	high
Röijezon et al. [71]	✓	X	NR	X	**✓**	**✓**	X	**X**	**✓**	**✓**	✓	NR	**X**	✓	11	moderate
Rutledge et al. [72]	✓	X	NR	X	**✓**	**X**	X	**✓**	**X**	**✓**	X	NR	**✓**	✓	10	moderate
Sarig Bahat et al. 2010 [73]	✓	X	NR	X	**X**	**X**	X	**✓**	**X**	**✓**	X	NR	**X**	✓	6	high
Sarig Bahat et al. 2015 [74]	✓	X	NR	X	**X**	**X**	X	**✓**	**X**	**✓**	X	NR	**✓**	✓	8	moderate
Sarig Bahat et al. 2020 [75]	✓	X	NR	NR	**✓**	**✓**	✓	**✓**	**✓**	**✓**	X	NR	**✓**	✓	15	low
Sjölander et al. [76]	✓	X	✓	X	**✓**	**✓**	X	**✓**	**✓**	**X**	X	NR	**✓**	✓	13	moderate
Takasaki et al. [77]	✓	X	✓	X	**x**	**X**	X	**✓**	**X**	**X**	X	NR	**✓**	✓	7	moderate
Tsang et al. 2013 [78]	✓	X	NR	X	**✓**	**✓**	X	**✓**	**✓**	**NR**	✓	NR	**✓**	✓	13	moderate
Tsang et al. 2014 [79]	✓	X	NR	X	**✓**	**✓**	X	**✓**	**✓**	**NR**	X	NR	**✓**	X	11	moderate
Vikne et al. [80]	✓	X	NR	X	**✓**	**X**	X	**✓**	**✓**	**✓**	✓	NR	**✓**	✓	13	moderate
Woodhouse et al. [81]	✓	X	NR	X	**X**	**X**	X	**✓**	**✓**	**✓**	✓	NR	**✓**	✓	11	moderate
Yang et al. [82]	✓	X	NR	X	**✓**	**X**	X	**✓**	**X**	**X**	X	NR	**X**	✓	6	high
Zhou et al. [83]	✓	x	NR	x	**✓**	**x**	x	X	**x**	**NR**	x	NR	**x**	✓	4	high

Rating: ✓ = Yes; x = No; NR = Not reported; RoB = Risk of Bias

^**a**^ implemented consistently across all study participants

**Items in bold:** have been weighted as being more crucial in assessing ROB and have therefore been counted twice

Total risk of bias: interpreted as **low** for score > 13, **moderate** for 7–13 and **high** for < 7 points

### Study characteristics

#### Publications and participants

All 27 included studies were cross-sectional studies, with sample sizes ranging from 20 to 167. Nine studies reported on INP exclusively [58, 59, 63, 66, 67, 71, 72, 78, 79], eight studies on WAD only [57, 61, 64, 68–70, 77, 80], six studies assessed outcomes for WAD and INP separately [60, 62, 65, 75, 76, 81], while another four studies reported on unclassified NP [73, 74, 82, 83]. Eight studies, that included patients after WAD [60, 69, 70, 73–77] did not report on a specific grade according to the Quebec Task classification [84], while another eight studies included patients after WAD grade I [61, 62, 64, 65, 68, 81], grade II [57, 61, 62, 64, 65, 68, 80, 81], or grade III [57, 61].

In total, 1′847 participants were examined across included studies, of which 911 were healthy controls, 631 participants with NP (INP and unclassified NP) and 305 participants with WAD. The average age of participants with NP ranged from 25.3 to 51.0 years; for participants with WAD from 27.0 to 49.0 years; and for controls from 19.9 to 50.0 years. Female participation rate dominated, with an average of 63% in the INP and unclassified NP, 72% in the WAD, and 58% in the control group. Average pain intensity, either reported as mean or median value, were transferred to a 0 to 100 measurement scale, and ranged from 20.1 to 60.0 for participants with INP and unclassified NP and from 29.0 to 66.1 for participants with WAD, likewise average disability, if provided, ranged from 9.5 to 37% for participants with INP and unclassified NP and from 25.2 to 45% for participants with WAD [57–83]. Table 4 provides further information on characteristics including studies’ criteria for being regarded as participant with NP or asymptomatic.

**Table 4:** Participants, demographics and clinical characteristics across included studies

Publication	Sample	Age (years) Mean (SD)	Pain intensity (0–100 scale) Mean (SD)	Symptom duration a priori defined	Authors’ definition of cases	Authors’ definition of controls regarding neck impairments
Baydal-Bertomeu et al. [57]	WAD 30 (15 F) C 29 (15 F)	20-50^a^ 20-50^a^	NA NA	> 6 months and < 1 year	WAD grade^b^ II and III with altered mobility of the neck	No WAD
Descarreaux et al. [58]	INP 19 (16 F) C 20 (13 F)	38.7 (10.7) 32.5 (8.8)	23 (20) NA	At least one episode of neck pain in the last 6 months	Suffering from non-traumatic chronic neck pain on a persistent or recurrent basis	Without any prior or current experience of spinal pain, cervicobrachial pain or other diffuse pain conditions
De Zoete et al. [59]	INP 50 (30 F) C 50 (27 F	35.5 (24.0–55.3)^a^ 34.5 (26.0–58.0)^a^	30 (18.77) NA	12 weeks or longer	Neck pain of at least 4/10 NRS (inquired date)	No current neck pain, had never sought treatment for neck pain
Ernst et al. [60]	INP 25 (14 F) WAD 13 (7 F) C 38 (21 F)	31.7 (12.03) 41.1 (11.4) 35.1 (13.1)	20.1 (21.5) 36.1 (17.8) 0 (0)	> 3 months	Traumatic or non-traumatic NP and a minimum Neck Disability Index score of 10%	No history of NP for which they sought treatment and a Neck Disability Index Score of less than 4%
Gadotti et al. [61]	WAD 5 (2 F) C 15 (10 F)	25.6 (4.9) 24.8 (1.9)	40 (20–60 ^a^ 3-6^e^) NA	NA	WAD grade^b^ I-III	No previous history of persistent neck pain, injury or visual problems
Grip et al. [62]	INP 21 (14 F) WAD 25 (17 F) C 24 (16 F)	49 (16) 49 (15) 50 (18)	49.2 (20.8) 66.1 (18.8) 0.5 (2.1)	Persistent symptoms	WAD: grade^b^ I and II INP: muscular symptoms without paraesthesia	Occasional neck or back pain accepted as long as free from symptoms in the past 3 months.
Hage et al. [63]	INP 9 (4 F) C 15 (3 F)	31 (14) 24 (3)	30 (0) 0 (0)	NA	3/10 NRS or grater and NDI > 8%	Absence of neck pain episodes in the last 6 months and NDI < = 8%
Kristjansson et al. [64]	WAD 20 (20 F) C 20 (20 F)	30.8 (9.1) 29.3 (8.6)	46.8 (21.8) NA	> 6 months and < 6 years	WAD grade^b^ I and II	No musculoskeletal pain or injury in the neck or upper limbs
Kristjansson and Oddsdottir [65]	INP 18 (11 F) WAD 18 (16 F) C 18 (8 F)	38.0 (8.3) 35.5 (11.9) 32.3 (10.9)	32 (15) /80 (14)^d^ 19 (14)/ 67 (26)^d^-	> 6 months and < 2 years	WAD: grade^b^ II after motor vehicle collision with no prior symptoms in the head or neck and pain score > 30 during last week INP: pain score > 30 during last week	No history of musculoskeletal pain or injury in the neck
Lemmers et al. [66]	INP 35 (19 F) C 100 (50 F)	48 (15) 44 (16)	40 (20) NA	NA	Cervical pain of unknown origin	Presence of non-specific neck pain at the time of invitation
Meisingset et al. [67]	INP 75 (55 F) C 91 (48 F)	43.1 (12.9) 40.8 (13.8)	46 (14) NA	Current neck pain episode lasting > 2 weeks	Non- traumatic neck pain as the main problem with a pain score of ≥30 at day of testing	No episode of neck pain within the last 3 months and no neck trauma
Oddsdottir et al. [68]	WAD 34 (28 F) C 31 (16 F)	42.1 (8.7) 37.9 (16.7)	30/78^d^ NA	> 6 months	WAD grade^b^ II with history of symptoms from the head or neck after 1 or more MVCs and pain intensity scoring > 40	No history of musculoskeletal pain or injury in the neck
Ohberg et al. [69]	WAD 59 (30 F) C 56 (27 F)	38.1 (10.6) 37.3 (10.9)	NA NA	> 3 months	Chronic WAD of different grades^b^	Without chronic head, neck or back pain Occasional neck or back pain accepted as long as free from symptoms during investigation.
Osterbauer et al. [70]	WAD 30 (25 F) C 51 (36 F)	27 (5) 24 (4)	47 (27)	9 (11) days^c^	Symptomatic whiplash-type neck trauma as a result of a rear end impact	No history of symptomatic neck trauma for which they sought treatment in the past year Neck disability index score less than 5%
Röijezon et al. [71]	INP 102 (102 F) C 33 (33 F)	INP 51 (9) C 47 (10)	54 (1.6) NA	> 3 months	Women with neck pain of non-traumatic origin with a decreased physical functioning measured as > 9 normalized points of the first 19 items in the Disability Arm Shoulder Hand questionnaire	Healthy women
Rutledge et al. [72]	INP 19 (5 F) C 22 (4 F)	27.5 (13.1) 19.9 (1.9)	46 (17) 0 (0)	–	Neck pain score above 30	Neck pain score 0 and symmetric lateral flexion regarding range and tissue resistance
Sarig Bahat et al. [73]	NP 25 (16 F): - INP 18 (NA) - WAD 7 (NA) C 42 (31 F)	39 (12.7) NA NA 35.3 (12.4)	33 (20) NA NA NA	> 6 weeks	Neck pain, either insidious or after whiplash injury, with or without referral to the upper limb	No history of spinal pain during the past year
Sarig Bahat et al. [74]	NP 33 (20 F): - INP 21 (NA) - WAD 12 (NA) C 22 (8 F)	37.5 (9.9) 33 (6.78)	36.4 (17.2) NA	> 3 months	Chronic neck pain with or without referral to the upper limb and neck range of motion more than 40° in each direction	No physical complaints in the neck region
Sarig Bahat et al. [75]	INP 12 (9 F)^f^ WAD: 8 (5 F)^f^ C 20 (10 F)	40.3 (9.8) 45.8 (16.5) 30.3 (6.2)^f^	NA	> 3 months	Absence of upper limb referral, and NDI > 10%	No history of neck pain in the last 3 months and NDI < 4%
Sjölander et al. [76]	INP 9 (9 F) WAD 7 (5 F) C 16 (13 F)	40 (9) 45 (11) 41 (9)	52 (16) 45 (19) NA	> 6 months	Neck pain	Absence of current, previous (over the last year) or repeated periods of neck pain
Takasaki et al. [77]	WAD14 (9 F) C 14 (8 F)	33.4 (10.8) 35.4 (10.7)	29 (16) NA	> 3 months and < 6 years	WAD subjects after a car accident with a score of greater than 8% on the Neck Disability Index	No history of a whiplash injury and no current neck pain or headache
Tsang et al. [78]	INP 34 (25 F) C 34 (25 F)	38.4 (10.8) 34.3 (9.0)	38.9 (15.8) NA	> 3 months or mostly presented over the last 12 months	Severity of neck condition had required medical care	No history of neck pain that required medical care over the last 12 months.
Tsang et al. [79]	INP 30 (22 F) C 30 (21 F)	38.3 (11.3) 35.1 (9.0)	37.6 (12.2) NA	> 3 months or mostly presented over the last 12 months	Severity of neck condition had required medical care	No history of neck pain that required medical care over the last 12 months
Vikne et al. [80]	WAD 15 (9 F) C 15 (9 F)	40.1 (8.7) 38.7 (8.8)	31 (14) NA	> 6 months	WAD grade^b^ II which started less than 72 h after the motor vehicle accident	No WAD
Woodhouse et al. [81]	INP 45 (32 F) WAD 35 (23 F) C 48 (24 F)	43 (32–53)^e^ 40 (32–48)^e^ 38 (27–48)^e^	40 (30–50)^e^ 60 (40–70)^e^ 0 (0–0)^e^	> 6 months and < 10 years	WAD: grade^b^ I and II suffering from NP and/or headache after a car collision with onset of symptoms within 48 h INP: no history of neck trauma	No previous or current neck pain or history of neck trauma
Yang et al. [82]	INP 18 (7 F) C 18 (8 F)	25.3 (4.6) 23.8 (3.9)	NA NA	NA-	Mechanical neck disorder diagnosed by a physician and who had sought medical treatment within the past 6 weeks	No history of cervical trauma, surgery or pain
Zhou et al. [83﻿]	INP 28 (16 F) C 23 (14 F)	45 (25–69) ^a^ 23 (23–30) ^a^	60 (20) NA	> 3 months	Mechanical or myofascial neck pain with at least one active trigger point in the cervico-thoracic or shoulder girdle region	Healthy university-aged participants

^a^ range, ^b^ WAD severity grade classification according to the Quebec Task Force Scale, ^c^ measured mean (SD), ^d^ minimal pain/ maximal pain mean (SD), ^e^ median value (interquartile range) ^f^ data obtained by authors

*C* controls, *F* female, *INP* idiopathic neck pain, *NA* no data available, *NP* unclassified neck pain (without description of onset mode), *NDI* neck disability index, *WAD* whiplash associated disorder

Symptom duration in seventeen studies lasted for at least 6 weeks or longer [57–60, 64, 65, 68, 70, 71, 73, 74, 76–79, 81, 83].

#### Movement tasks examined in included studies (Table 5)

The movement tasks used to assess kinematics were either *head-aiming* [58–61, 63–65, 67, 68, 70, 73–75, 81]*, functional* [62, 77, 79]*,* or *unconstrained tasks* [57, 62, 66, 67, 69, 71, 72, 76, 78, 80, 82, 83]. Movement accuracy was assessed exclusively by *head aiming* tasks [58–61, 63–65, 67, 68, 70, 73–75, 81].

**Table 5:** Movement tasks and further specifications

Tasks		Specified Outcome variables	Task specifications	
Head aiming (*n* = 14)	Tracking (*n* = 10)	Velocity variables (n = 2) Temporal variables ((*n* = 2) Movement smoothness (*n =* 2) Movement accuracy (*n* = 8)	Starting position Repetitions	Tracking cursor speed Tracking pattern Track predictable/ unpredictable
	Pointing (*n* = 5)	Velocity variables (*n* = 3) Acceleration variables (*n =* 1) Temporal variables (*n* = 4) Movement smoothness (*n =* 2)		Target size Target speed Target direction Target predictable/ unpredictable
Functional (*n =* 3)		Velocity variables (*n =* 2) Acceleration variables (*n =* 1)		Task predictable/ unpredictable Task speed
Unconstrained (*n* = 12)		Velocity variables (*n* = 10) Acceleration variables (*n =* 4) Temporal variables (*n =* 5) Movement smoothness (*n* = 7)		Cyclic/ single motion Motion direction Eyes open/ closed Speed instruction Motion range instruction

(*n =* number of studies)

During *head-aiming tasks*, participants wore a head-mounted device that projected a visible point on a screen or wall in front of them. They controlled the position of that signal by moving their head either accurately along a trajectory *(tracking)* [59, 60, 64, 65, 67, 68, 70, 73–75, 81], or towards a target point *(pointing)* [58, 61, 63, 73–75]*.* These target points and trajectory paths were either visible before and during the tasks (predictable) [58, 60, 63, 70, 79, 81] or appeared unpredictably (unpredictable) [61, 64, 65, 68, 73, 74, 77].

Three studies examined head kinematics while participants performed *functiona*l *tasks*, such as driving in a simulator, catching a ball, or lifting a weight [62, 77, 79]. During *unconstrained* movement tasks, participants were asked to move their head in a specific direction like in rotation or extension [57, 62, 66, 67, 69, 71, 72, 76, 78, 80, 82, 83]. Further tasks specifications were used in relation to speed, repetitions, or the amplitude of movement. (See Table 5).

#### Measurement devices

To assess kinematics, electromagnetic motion tracking systems were used in thirteen studies [59, 64–68, 71, 73, 78–82], optical motion capture systems in eight studies [57, 58, 61–63, 69, 70, 72], virtual reality tracking systems in three studies [73–75], and inertial motion capture systems in another three studies [74, 77, 83].

For movement accuracy assessments, five studies used an electromagnetic tracking system [59, 64, 65, 67, 68], two studies a virtual reality tracking system [74, 75] and two studies a head-mounted laser pointer [60, 75].

#### Outcome measures

Table 6 provides an overview of specified outcome measures and variables reported in included studies. Five groups of outcome measures emerged that describe different sensorimotor control alterations related to NP.

**Table 6:** Summary of outcome measures

Outcome measure	No. of studies	Specified outcome variable	No. of studies
Velocity	18	Mean velocity	10
		Peak velocity	12
		Normalized peak amplitude	1
Acceleration	6	Mean acceleration	1
		Peak acceleration	4
		Peak deceleration	2
		Magnitude of circumduction vector	1
Temporal	11	Movement time	6
		Reaction time	3
		Acceleration phase duration	1
		Deceleration phase duration	1
		Ratio of phase durations	4
Movement smoothness	11	Normalized jerk cost	4
		Root mean square jerk	1
		Number of jerk peaks	1
		Root mean square velocity	1
		Number of velocity peaks	2
		Speed index of deviation	1
		Spectral entropy	1
		Harmonicity	1
Movement accuracy	8	Number of errors	2
		Point deviation	7
		Time on target	2

*Velocity* and *acceleration measures* were assessed in eighteen studies and summarised as discrete or continuous variables calculated either from velocity [57, 62, 63, 67, 69–80, 82] or acceleration time series [57, 63, 78–80, 83].

*Temporal measures investigated in eleven studies* incorporated time-related variables and were calculated as duration of different phases of a movement such as acceleration or deceleration [58], as ratio of phase durations [71, 73, 74, 80], and in addition as time to complete [58, 60, 61, 75, 82, 83], or to initiate a task [61, 69, 73].

*Movement smoothness* measured in eleven studies [57, 66, 68, 71, 73, 74, 76, 80–83], considered the degree of interruptions affecting the continuous and smooth evolvement of a movement. Most of the variables used were velocity or jerk-based; of these, some were dimensionless, which means. Independent of the movement amplitude and duration, such as the normalized jerk cost [68, 76, 80, 83], while others were not, like the root mean square jerk [66]. Speed index of deviation quantified the degree to which a movement’s speed was optimised, to minimise jerk [71]. Other variables of movement smoothness measured the complexity found in a movement, with complexity reflecting how a movement evolves from a series of sub-movements [57]. Spectral entropy, which measures the complexity in the power spectrum of a movement, was also used for this purpose [82].

*Movement accuracy* measures used in eight studies [59, 60, 64, 65, 67, 68, 74, 75] described the proximity of a movement to a given target area or target trajectory and outcome variables used were counting the numbers of errors while following a given trajectory [60, 75], calculating the point deviation from a tracking path [59, 64, 65, 67, 68, 74, 75], and measuring the time the trajectory remained on a target [65, 68].

### Summary of findings

Clinical and methodological heterogeneity regarding participant characteristics, task specifications, and kinematic or movement accuracy outcome variables was large for all studies. Consequently, findings were summarized qualitatively only, and are presented in Tables 7, 8, 9, 10 and 11. All results are presented for NP and its subgroups (INP or WAD), when compared to control participants.

**Table 7:** Outcome Summary for Velocity variables

**Mean Velocity**					
*Summary of findings for NP/ INP:* • *Inconsistent findings on mean velocity in NP/ INP patients compared to healthy controls* • *Level of evidence:* ***conflicting*** *Summary of findings for WAD:* • *Decreased mean velocity in WAD compared to healthy controls* • *Level of evidence:* ***moderate***					
**Publication**	**Sample**	**Task**	**Task specifications**	**Results compared to C**	**Risk of Bias**
Sarig Bahat et al. [75]	NP 20 C 20	Head aiming; Tracking	Speed: Self-preferred Tracking path: Predictable Pattern: Zig Zag	Decreased in NP	Low
Grip et al. [62]	WAD 21 INP 25 C 24	Unconstrained	Speed: As fast as possible Directions: FLEX/EXT, ROT	Decreased in WAD Decreased in INP	Moderate
		Functional	Speed: Given Task: unpredictable Function: catching a ball with both hands at left or right shoulder height	Decreased in WAD No differences in INP	
Ohberg et al. [69]	WAD 59 C 56	Unconstrained	Speed: As fast as possible Directions: FLEX/EXT, ROT	Decreased in WAD	Moderate
Rutledge et al. [72]	INP 19 C 22	Unconstrained	Speed: Slow Directions: LFLEX	No significant differences	Moderate
Sarig Bahat et al. [74]	NP 33 C 22	Head aiming; Pointing	Speed: As fast as possible Targets: Unpredictable Directions: FLEX/EXT, ROT	Decreased in NP	Moderate
Tsang et al. [78]	INP 34 C 34	Unconstrained	Speed: Self-preferred Directions: FLEX/EXT, ROT, LFLEX	Decreased in INP	Moderate
Vikne et al. [80]	WAD 15 C 15	Unconstrained	Speed: Slow(S), preferred (P), max (MAX) Directions: FLEX/EXT	Decreased in WAD for EFN and FBN in S and P Decreased in WAD for all directions in MAX	Moderate
Hage et al. [63]	INP 9 C 15	Head aiming; Pointing	Speed: As fast as possible Targets: Predictable Directions: ROT	No significant differences	High
Osterbauer et al. [70]	WAD 30 C 51	Head aiming; Tracking	Speed: Self-preferred Tracking path: Predictable Pattern: Vertical line	Decreased in WAD	High
Sarig Bahat et al. [73]	NP 25 C 42	Head aiming; Pointing	Speed: As fast as possible Targets: Unpredictable Directions: FLEX/EXT, ROT	Decreased in NP	High
**Peak Velocity**					
*Summary of findings for NP/ INP:* *• Inconsistent findings on peak velocity in NP/ INP patients compared to healthy controls* *• Level of evidence:* ***conflicting*** *Summary of findings for WAD:* *• Inconsistent findings on peak velocity in WAD patients compared to healthy controls* *• Level of evidence:* ***conflicting***					
**Publication**	**Sample**	**Task**	**Task specifications**	**Results compared to C**	**Risk of Bias**
Baydal-Bertomeu et al. [57]	WAD 30 C 29	Unconstrained	Speed: Self-preferred Direction: FLEX/EXT	Decreased in WAD	Moderate
Meisingset et al. [67]	INP 75 C 91	Unconstrained	Speed: Self-preferred Directions: FLEX/EXT, ROT, LFLEX	Decreased in INP	Moderate
Ohberg et al. [69]	WAD 59 C 56	Unconstrained	Speed: As fast as possible Directions: FLEX/EXT, ROT	Decreased in WAD	Moderate
Röijezon et al. [71]	INP 118 C 51	Unconstrained	Speed: As fast as possible Direction: ROT	Decreased in INP	Moderate
Sarig Bahat et al. [74]	NP 33 C 22	Head aiming; Pointing	Speed: As fast as possible Targets: Unpredictable Directions: FLEX/EXT, ROT	Decreased in NP	Moderate
Sjölander et al. [76]	WAD 7 INP 9 C16	Unconstrained	Speed: As fast as possible Direction: ROT	No significant differences	Moderate
Takasaki et al. [77]	WAD 14 C 14	Functional	Speed: Self-preferred Task: Unpredictable Function: Driving simulator in 3 different traffic scenarios	No significant differences	Moderate
Tsang et al. [79]	INP 30 C 30	Functional	Speed: Self- preferred Task: Predictable Function: Lifting a 2 kg weight by one hand from a desk to a shelf	Decreased in INP	Moderate
Vikne et al. [80]	WAD 15 C 15	Unconstrained	Speed: Slow(S), preferred (P), max (MAX) Directions: FLEX/EXT	Decreased in WAD for EFN and FBN in S and P Decreased in WAD for all directions in MAX	Moderate
Hage et al. [63]	INP 9 C 15	Head aiming; Pointing	Speed: As fast as possible Targets: Predictable Directions: ROT	No significant differences	High
Sarig Bahat et al. [73]	NP 25 C 42	Head aiming; Pointing	Speed: As fast as possible Targets: Unpredictable Directions: FLEX/EXT, ROT	Decreased in NP	High
Yang et al. [82]	NP 18 C 18	Unconstrained	Speed: Self-preferred Direction: Circumduction	No significant differences	High
**Normalized Peak Amplitude**					
*Summary of findings for INP:* *• No difference on normalized peak amplitude in INP patients compared to healthy controls* *• Level of evidence:* ***limited***					
**Publication**	**Sample**	**Task**	**Task specifications**	**Results compared to C**	**Risk of Bias**
Röijezon et al. [71]	INP 118 C 51	Unconstrained	Speed: As fast as possible Direction: ROT	No significant differences	Moderate

*C* controls, *EFN* Extension from neutral position, *EXT* Extension, *F* fast, *FBN* Flexion back to neutral position, *FLEX* Flexion, *INP* idiopathic neck pain, *LFLEX* Lateralflexion, *MAX* maximal, *NP* unclassified neck pain, *P* preferred, *ROT* Rotation, *S* slow, *WAD* whiplash associated disorder

**Table 8:** Outcome Summary for Acceleration variables

**Mean Acceleration**					
*Summary of findings for INP:* *• Decreased mean acceleration in INP patients compared to healthy controls* *• Level of evidence:* ***limited***					
**Publication**	**Sample**	**Task**	**Task specifications**	**Results compared to C**	**Risk of Bias**
Tsang et al. [78]	INP 34 C 34	Unconstrained	Speed: Self-preferred Directions: FLEX/EXT, ROT, LFLEX	Decreased in INP	moderate
**Peak Acceleration**					
*Summary of findings for INP:* *• Inconsistent findings on peak acceleration in INP patients compared to healthy controls* *• Level of evidence:* ***conflicting*** *Summary of findings for WAD:* *• Decreased peak acceleration in WAD patients compared to healthy controls* *• Level of evidence:* ***moderate***					
**Publication**	**Sample**	**Task**	**Task specifications**	**Results compared to C**	**Risk of Bias**
Baydal-Bertomeu et al. [57]	WAD 30 C 29	Unconstrained	Speed: Self-preferred Direction: FLEX/EXT	Decreased in WAD	moderate
Tsang et al. [79]	INP 30 C 30	Functional	Speed: Self- preferred Task: Predictable Function: Lifting a 2 kg weight by one hand from a desk to a shelf	Decreased in INP	moderate
Vikne et al. [80]	WAD 15 C 15	Unconstrained	Speed: Slow(S), preferred (P), max (MAX) Directions: FLEX/EXT	Decreased in WAD for FBN in S Decreased in WAD for EFN and FBN in P Decreased in WAD for all directions in MAX	moderate
Hage et al. [63]	INP 9 C 15	Head aiming; Pointing	Speed: as fast as possible Targets: predictable Directions: ROT	No significant differences	high
**Peak Deceleration**					
*Summary of findings for INP:* *• No difference on peak deceleration in INP patients compared to healthy controls* *• Level of evidence:* ***very limited*** *Summary of findings for WAD:* *• Decreased peak deceleration in WAD patients compared to healthy controls in maximal speed condition* *• Level of evidence:* ***limited***					
Vikne et al. [80]	WAD 15 C 15	Unconstrained	Speed: Slow(S), preferred (P), max (MAX) Directions: FLEX/EXT	Decreased in WAD for EFN in S Decreased in WAD for EFN and FBN in P Decreased in WAD for all directions in MAX	moderate
Hage et al. [63]	INP 9 C 15	Head aiming; Pointing	Speed: as fast as possible Targets: predictable Directions: ROT	No significant differences	high
**Magnitude of Circumduction Vectors (MCV)**					
*Summary of findings for NP:* *• Decreased MCV in NP patients compared to healthy controls* *• Level of evidence:* ***very limited***					
**Publication**	**Sample**	**Task**	**Task specifications**	**Results compared to C**	**Risk of Bias**
Zhou et al. [83]	NP 28 C 23	Unconstrained	Speed: Self-preferred Direction: Circumduction	Decreased in NP	high

*C* controls, *EFN* Extension from neutral position, *EXT* Extension, *F* fast, *FBN* Flexion back to neutral position, *FLEX* Flexion, *INP* idiopathic neck pain, *LFLEX* Lateralflexion, MAX maximal, *MCV* Magnitude of circumduction vector, *NP* unclassified neck pain, *P* preferred, *ROT* Rotation, *S* slow, *WAD* whiplash associated disorder

**Table 9:** Outcome Summary for Temporal Variables

**Movement Time**					
*Summary of findings for NP/ INP:* *• Increased movement time in NP/ INP patients compared to healthy controls* *• Level of evidence:* ***strong*** *Summary of findings for WAD:* *• Inconsistent findings on movement time in WAD patients compared to healthy controls* *• Level of evidence:* ***conflicting***					
**Publication**	**Sample**	**Task**	**Task specifications**	**Results compared to C**	**Risk of Bias**
Ernst et al. [60]	INP 25 WAD 13 C 38	Head aiming; Tracking	Speed: Self-preferred Tracking Path: Predictable Patterns: Figure of eight (F8), Zig Zag (ZZ)	Increased in INP No significant differences in WAD	low
Sarig Bahat et al. [75]	INP 12 WAD 8 C 20	Head aiming; Tracking	Speed: Self-preferred Tracking Path: Predictable Pattern: Zig Zag (ZZ)	Increased in INP No significant differences in WAD	low
Descarreaux et al. [58]	INP 19 C 20	Head aiming; Pointing	Speed: As fast as possible Target: Predictable Direction: ROT	Increased in INP	moderate
Gadotti et al. [61]	WAD 5 C 15	Head aiming; Pointing	Speed: As fast as possible Target: Unpredictable Direction: ROT	Increased in WAD	moderate
Yang et al. [82]	NP 18 C 18	Unconstrained	Speed: Self-preferred Direction: Circumduction	Increased in NP	high
Zhou et al. [83]	NP 28 C 23	Unconstrained	Speed: Self-preferred Direction: Circumduction	Increased in NP	high
**Reaction Time**					
*Summary of findings for NP:* *• No differences on reaction time in NP patients compared to healthy controls* *• Level of evidence:* ***very limited*** *Summary of findings for WAD:* *• Increased reaction time in WAD patients compared to healthy controls* *• Level of evidence:* moderate*					
**Publication**	**Sample**	**Task**	**Task specifications**	**Results compared to C**	**Risk of Bias**
Gadotti et al. [61]	WAD 5 C 15	Head aiming; Pointing	Speed: As fast as possible Target: Unpredictable Direction: ROT	Increased in WAD	moderate
Ohberg et al. [69]	WAD 59 C 56	Unconstrained	Speed: As fast as possible Directions: FLEX/EXT, ROT	Increased in WAD	moderate
Sarig Bahat et al. [73]	NP 25 C 42	Head aiming; Pointing	Speed: As fast as possible Targets: Unpredictable Directions: FLEX/EXT, ROT	No significant differences	high
**Acceleration Phase Duration**					
*Summary of findings for INP:* *• No differences in acceleration phase duration in NP patients compared to healthy controls* *• Level of evidence:* ***limited***					
**Publication**	**Sample**	**Task**	**Task specifications**	**Results compared to C**	**Risk of Bias**
Descarreaux et al. [58]	INP 19 C 20	Head aiming; Pointing	Speed: As fast as possible Target: Predictable Direction: ROT	No significant differences	moderate
**Deceleration Phase Duration**					
*Summary of finding:* *• Increased deceleration phase duration in INP patients compared to healthy controls* *• Level of evidence:* ***limited***					
**Publication**	**Sample**	**Task**	**Task specifications**	**Results compared to C**	**Risk of Bias**
Descarreaux et al. [58]	INP 19 C 20	Head aiming; Pointing	Speed: As fast as possible Target: Predictable Direction: ROT	Increased in NP	moderate
**Ratio of Phase Durations**					
*Summary of findings for NP/ INP:* *• Inconsistent findings on ratio of phase durations in NP/ INP patients compared to healthy controls* *• Level of evidence:* ***conflicting*** *Summary of findings for WAD:* *• No difference on ratio of phase durations in WAD patients compared to healthy controls* *• Level of evidence:* ***limited***					
**Publication**	**Sample**	**Task**	**Task specifications**	**Results compared to C**	**Risk of Bias**
Röijezon et al. [71]	INP 118 C 51	Unconstrained	Speed: As fast as possible Direction: ROT	No significant differences	Moderate
Sarig Bahat et al. [74]	NP 33 C 22	Head aiming; Pointing	Speed: As fast as possible Targets: Unpredictable Directions: FLEX/EXT, ROT	Decreased in NP except for target in LROT	Moderate
Vikne et al. [80]	WAD 15 C 15	Unconstrained	Speed: Slow(S), Preferred (P), Max (MAX) Direction: FLEX/EXT	No significant differences	Moderate
Sarig Bahat et al. [73]	NP 25 C 42	Head aiming; Pointing	Speed: As fast as possible Targets: Unpredictable Directions: FLEX/EXT, ROT	No significant differences	High

*C* controls, *EXT* Extension, *F8* Figure of eight, *FLEX* Flexion, *INP* idiopathic neck pain, *MAX* maximal, *NP* unclassified neck pain, *P* preferred, *ROT* Rotation, *S* slow, *WAD* whiplash associated disorder, *ZZ* Zig Zag

**Table 10:** Outcome Summary for Movement Smoothness Variables

**Normalized Jerk Cost**					
*Summary of findings for NP/ INP:* *• Inconsistent findings on normalized jerk cost in NP/ INP patients compared to healthy controls* *• Level of evidence:* ***conflicting*** *Summary of findings for WAD:* *• Inconsistent findings on normalized jerk cost in WAD patients compared to healthy controls* *• Level of evidence:* ***conflicting***					
**Publication**	**Sample**	**Task**	**Task specifications**	**Results compared to C**	**Risk of Bias**
Oddsottir et al. [68]	WAD 34 C 31	Head aiming; Tracking	Speed: Given for the target cursor Tracking path: Unpredictable Patterns: 3 incremental difficulties	Increased in WAD for the easy and medium difficult pattern	moderate
Sjölander et al. [76]	WAD 7 INP 9 C16	Unconstrained	Speed: As fast as possible Direction: ROT	Increased in INP for In-Left and Out-Left Increased in WAD for Out-Left	moderate
Vikne et al. [80]	WAD 15 C 15	Unconstrained	Speed: Slow(S), preferred (P), max (MAX) Directions: FLEX/EXT	No significant differences	moderate
Zhou et al. [83]	NP 28 C 23	Unconstrained	Speed: Self-preferred Direction: Circumduction	No significant differences	high
**Root Mean Square Jerk**					
*Summary of findings for INP:* *• No differences on root mean square jerk in INP patients compared to healthy controls* *• Level of evidence:* ***limited***					
Lemmers et al. [66]	INP 35 C 100	Unconstrained	Speed: Self-preferred Direction: LFLEX	No significant differences	moderate
**Number of Jerk Peaks**					
*Summary of findings for NP:* *• Increased number of jerk peaks in NP patients compared to healthy controls* *• Level of evidence:* ***very limited***					
**Publication**	**Sample**	**Task**	**Task specifications**	**Results compared to C**	**Risk of Bias**
Zhou et al. [83]	NP 28 C 23	Unconstrained	Speed: Self-preferred Direction: Circumduction	Increased in NP	high
**Root Mean Square Velocity**					
*Summary of findings for INP:* *• No difference on root mean square velocity in INP patients compared to healthy controls* *• Level of evidence:* ***limited*** *Summary of findings for WAD:* *• Increased root mean square velocity in WAD patients compared to healthy controls in slow and moderate speed conditions* *• Level of evidence:* ***limited***					
**Publication**	**Sample**	**Task**	**Task specifications**	**Results compared to C**	**Risk of Bias**
Woodhouse et al. [81]	WAD 35 INP 45 C 48	Head aiming; Tracking	Speed: Slow (S), moderate (MOD), fast (F) Tracking path: Predictable Pattern: Figure of eight	Increased in WAD for S and MOD No significant differences in INP	Moderate
**Number of Velocity Peaks**					
*Summary of findings for NP:* *• Inconsistent findings on number of velocity peaks in NP patients compared to healthy controls* *• Level of evidence:* ***conflicting***					
**Publication**	**Sample**	**Task**	**Task specifications**	**Results compared to C**	**Risk of Bias**
Sarig Bahat et al. [74]	NP 33 C 22	Head aiming; Pointing	Speed: As fast as possible Targets: Unpredictable Directions: FLEX/EXT, ROT	Increased in NP	Moderate
Sarig Bahat et al. [73]	NP 25 C 42	Head aiming; Pointing	Speed: As fast as possible Targets: Unpredictable Directions: FLEX/EXT, ROT	Decreased in NP	High
**Speed Index of Deviation**					
*Summary of findings for INP:* *• Increased speed index of deviation in INP patients compared to healthy controls* *• Level of evidence:* ***limited***					
**Publication**	**Sample**	**Task**	**Task specifications**	**Results compared to C**	**Risk of Bias**
Röijezon et al. [71]	INP 118 C 51	Unconstrained	Speed: As fast as possible Direction: ROT	Increased in INP	moderate
**Spectral Entropy**					
*Summary of findings:* *• Increased spectral entropy in NP patients compared to healthy controls* *• Level of evidence:* ***very limited***					
**Publication**	**Sample**	**Task**	**Task specifications**	**Results compared to C**	**Risk of Bias**
Yang et al. [82]	NP 18 C 18	Unconstrained	Speed: Self-preferred Direction: Circumduction	Increased in NP	high
**Harmonicity**					
*Summary of findings for WAD:* *• No significant differences on harmonicity in WAD patients compared to healthy controls* *• Level of evidence:* ***limited***					
**Publication**	**Sample**	**Task**	**Task specifications**	**Results compared to C**	**Risk of Bias**
Baydal-Bertomeu et al. [57]	WAD 30 C 29	Unconstrained	Speed: Self-preferred Direction: FLEX/EXT	No significant differences	moderate

*C* controls, *EXT* Extension, *F* fast,, *FLEX* Flexion, *INP* idiopathic neck pain, *LFLEX* Lateralflexion, *MAX* maximal, *MOD* moderate, *NP* unclassified neck pain, *P* preferred, *ROT* Rotation, *S* slow, *WAD* whiplash associated disorder

**Table 11:** Outcome Summary for Movement Accuracy Variables

**Number of Errors**					
*Summary of findings for INP:* *• Increased number of errors in INP patients compared to healthy controls* *• Level of evidence:* ***strong*** *Summary of findings for WAD:* *• Increased number of errors in WAD patients compared to healthy controls* *• Level of evidence:* ***strong***					
**Publication**	**Sample**	**Task**	**Task specifications**	**Results compared to C**	**Risk of Bias**
Ernst et al. [60]	INP 25 WAD 13 C 38	Head aiming; Tracking	Speed: Self-preferred Tracking Path: Predictable Patterns: Figure of eight (F8), Zig Zag (ZZ)	Increased in INP Increased in WAD	low
Sarig Bahat et al. [75]	INP 12 WAD 8 C 20	Head aiming; Tracking	Speed: Self-preferred Tracking Path: Predictable Pattern: Zig Zag (ZZ)	Increased in INP Increased in WAD	low
**Point Deviation**					
*Summary of findings in NP/ INP:* *• Inconsistent findings on point deviation in NP/ INP patients compared to healthy controls* *• Level of evidence:* ***conflicting*** *Summary of findings in WAD:* *• Increased point deviation in WAD patients compared to healthy controls* *• Level of evidence:* ***moderate***					
**Publication**	**Sample**	**Task**	**Task specifications**	**Results compared to C**	**Risk of Bias**
Sarig Bahat et al. [75]	INP 12 WAD 8 C 20	Head aiming; Tracking	Speed: Given for the target cursor Tracking Path: Unpredictable Pattern: Horizontal and vertical line	Increased in INP for horizontal directions Increased in WAD for all directions	low
De Zoete et al. [59]	INP 50 C 50	Head aiming; Tracking	Speed: Given for the target cursor Tracking path: Unpredictable	No significant differences	low
Kristjansson et al. [64]	WAD 20 C 20	Head aiming; Tracking	Speed: Given for the target cursor Tracking path: Unpredictable	Increased in WAD	moderate
Kristjansson and Oddsdottir et al. [65]	WAD 18 INP 18 C 18	Head aiming; Tracking	Speed: Given for the target cursor Tracking path: Unpredictable	Increased in WAD Increased in INP	moderate
Meisingset et al. [67]	INP 75 C 91	Head aiming; Tracking	Speed: Given for the target cursor, low speed and high speed A) Tracking path: Predictable Pattern: Figure of eight B) Tracking path: Unpredictable Patterns: Two incremental difficulties	Decreased in INP in high speed sitting and low speed standing in A) Decreased in INP for the easy pattern in B)	moderate
Oddsottir et al. [68]	WAD 34 C 31	Head aiming; Tracking	Speed: Given for the target cursor Tracking path: Unpredictable	Increased in WAD	moderate
Sarig Bahat et al. [74]	NP 33 C 22	Head aiming; Tracking	Speed: Given for the target cursor Tracking Path: Unpredictable Pattern: Horizontal and vertical line	Increased in NP	moderate
**Time on Target**					
*Summary of findings for INP:* *• Decreased time on target in INP patients compared to healthy controls* *• Level of evidence:* ***limited*** *Summary of findings for WAD:* *• Decreased time on target in WAD patients compared to healthy controls* *• Level of evidence:* ***moderate***					
**Publication**	**Sample**	**Task**	**Task specifications**	**Results compared to C**	**Risk of Bias**
Kristjansson and Oddsdottir et al. [65]	WAD 18 INP 18 C 18	Head aiming; Tracking	Speed: Given for the target cursor Tracking path: Unpredictable	Decreased in WAD Decreased in INP	moderate
Oddsottir et al. [68]	WAD 34 C 31	Head aiming; Tracking	Speed: Given for the target cursor Tracking path: Unpredictable	Decreased in WAD	moderate

*C* controls, *F8* Figure of eight, *INP* idiopathic neck pain, *NP* unclassified neck pain, *WAD* whiplash associated disorder, *ZZ* Zig Zag

#### Velocity variables (Table 7)

Ten studies assessed *mean velocity* of head motion in participants as outcome variable [62, 63, 69, 70, 72–75, 78, 80]. Seven studies on INP or unclassified NP showed a **conflicting** level of evidence [62, 63, 72–75, 78]. In contrast, those four studies on WAD subjects only, showed a decrease in *mean velocity*, and resulted in a **moderate** level of evidence [62, 69, 70, 80]. Twelve studies on *peak velocity* demonstrated a **conflicting** level of evidence for all NP groups [57, 63, 67, 69, 71, 73, 74, 76, 77, 79, 80, 82], with four studies of moderate and high RoB, indicating no differences in *peak velocity* in NP [63, 76, 77, 82], while, in contrast to these, eight studies described a decreased *peak velocity* in NP [57, 67, 69, 71, 73, 74, 79, 80]. One study presented a ratio of peak to mean velocity *(normalized peak amplitude)* in INP, and found no differences [71].

#### Acceleration variables (Table 8)

One study of moderate quality looked at *mean acceleration* and found that patients with INP showed a reduced acceleration, that resulted in a **limited** level of evidence [78]. Two studies were investigating *peak acceleration* in INP, and showed inconsistent results, leading to a **conflicting** level of evidence [63, 79]. While another two studies that focused on patients with WAD showed *peak acceleration* to be lowered [57, 80]. Two studies were assessing *peak deceleration* which led to a **very limited** level of evidence for INP to move with similar [63], and a **limited** level of evidence for WAD to move with decreased *peak deceleration* [80]. A **very limited** level of evidence was found for a decreased *Magnitude of Circumduction* vectors, as one study of high RoB assessed this outcome variable [83].

Across all acceleration variables and NP groups, *unconstrained movement tasks* showed a **moderate** level of evidence of being reduced [57, 78, 80, 83], while for *functional* or *head aiming task* such an effect could not be demonstrated [63, 79].

#### Temporal variables (Table 9)

Six studies examined the *movement time* needed to complete a movement task [58, 60, 61, 75, 82, 83]. In five of these studies and for INP and unclassified NP, a **strong** level of evidence for an increased *movement time* was found [58, 60, 75, 82, 83]. In contrast, three studies on WAD, showed inconsistent findings, that lead to a **conflicting** level of evidence [60, 61, 75]. Three studies looked at the *reaction time* to initiate a movement task [61, 69, 74]. A **moderate** level of evidence could be demonstrated for an increased *reaction time* in WAD [61, 69], while for unclassified NP a **very limited** evidence for no differences was found by one study with a high RoB [73]. One study provided a **limited** level of evidence for an increased *deceleration phase* and no differences in *acceleration phase* duration in patients with INP [58]. Four studies examined a *ratio of phase duration* and provided **limited** level of evidence for WAD [80], and **conflicting** level of evidence for INP and unclassified NP [71, 73, 74].

#### Movement smoothness: (Table 10)

Eleven studies examined eight outcome variables and demonstrated a **limited**, **very limited** or **conflicting** level of evidence [57, 66, 68, 71, 73, 74, 76, 80–83]. A **limited** level of evidence could be demonstrated for an increased *spectral entropy* in unclassified NP [82], an increased *speed index of deviation* in INP [71], and an increased *root mean square velocity* in WAD [81]. Furthermore, a **limited** level of evidence of no differences in INP could be found for *root mean square jerk* [66] and *root mean square velocity* [81]. A **very limited** level of evidence was found for an increased *number of jerk peaks* [83] and *spectral entropy* [82] in unclassified NP. A **conflicting** level of evidence existed for *normalized jerk cost*, for all NP subgroups [68, 76, 80, 83], and for the *number of velocity peaks* in unclassified NP [73, 74].

#### Movement accuracy (Table 11)

Movement accuracy was assessed in eight studies and on three outcome variables [59, 60, 64, 65, 67, 68, 74, 75]. A **strong** level of evidence was found for an increased *number of errors* in INP and WAD [60, 75]. A **moderate** level of evidence was found for an increased *point deviation* [59, 64, 65, 67, 68, 74, 75] and a decreased *time on target* in WAD [65, 68]. A **limited** level of evidence was demonstrated for decreased *time on target* in INP [65]. A **conflicting** level of evidence was found for *point deviation* in unclassified NP [65, 67, 74, 75].

## Discussion

This systematic review aimed to collate various movement tasks and outcome variables, that had been used to examine time-domain related head kinematics and movement accuracy in case control studies comparing patients with NP with asymptomatic controls. Three different movement tasks were employed, head aiming towards a target, performing functional tasks, or moving the head without constraints, as in circumduction or rotation. Strong evidence was found for movement time being increased during the performance of a movement task, such as head tracking, pointing, or unconstrained head movements in patients with NP. Furthermore, there was strong evidence of decreased movement accuracy, in terms of an increased number of errors made during a head tracking task in INP and WAD, when compared to control participants. The latter were only examined in two studies, both demonstrated a low RoB, while the former was investigated in six studies with low to high RoB. Moderate evidence was detected showing decreased mean velocity, a decreased peak acceleration, decreased time on target, increased point deviation and reaction time for patients with WAD compared to healthy controls. In addition, a moderate level of evidence has been found for all acceleration variables during unconstrained movement tasks. Other kinematic and/or movement accuracy variables demonstrated only limited, very limited, or even, conflicting results.

Some of the findings suggest impaired sensorimotor control in NP in respect to their kinematic and movement accuracy abilities, while other findings were dependent on the specific NP subgroup investigated or the specific outcome variable assessed. The overall methodological quality, or risk of bias, of included studies was moderate, as many studies did not provide adequate information to prevent bias, such as selection bias, blinding of study personnel, and confounding, e.g., not matching for age or sex (see Table 3).

Studies showed high clinical and methodological variability. Clinical variability was shown particularly through differences in the definition of the NP status, while methodological variability was found with respect to the movement tasks, including specifications and defined outcomes. These issues, together with the overall RoB, imply that the results of this review should be interpreted with caution, since comparability was limited and accordingly pooling of results for quantitative analysis was not possible.

In addition, all included studies are case-control studies within cross-sectional designs, which limits their generalisability and diagnostic accuracy implied by the potential selection bias within this study design itself [85].

Findings on movement accuracy in patients with WAD were robust, independent of outcome variables used, as patients with WAD showed a reduced movement accuracy with moderate to strong level of evidence. The same seems to be true for movement time in INP and unclassified NP, though not for WAD. However, as time and accuracy within a head aiming task are usually regarded inversely related, known as the speed-accuracy trade-off [86], both outcome measures must be regarded in combination [87]. Accordingly, this association needs to be accounted for in those studies that found differences in movement accuracy measures, but could not, for the same test, demonstrate differences in movement time [60, 75]. These findings give an indication that patients with WAD may prefer speed to accuracy as a movement strategy, if the task allows for. Unconstrained movement tasks were most frequently used in studies to determine acceleration outcomes [57, 78, 80, 83] and have demonstrated a moderate level of evidence for a decreased acceleration in NP. It seems that, irrespective of further movement specifications, such as speed or displacement, patients with NP differ from controls for acceleration variables. A main effect for other movement tasks on other outcome measures could not be determined. Though, within those three studies that used functional tasks, only velocity and acceleration variables have been examined [62, 77, 79]. Inconsistent or opposing results for some outcome variables, led to conflicting levels of evidence within in our review. Some of these may be explained by insufficient sample sizes to determine a group difference (type II error). This may have occurred for mean-, peak velocity, and movement time, as all studies, that found no differences between NP and controls had sample sizes below twenty [60, 72, 77, 82] or even below ten [63, 76] for their NP groups. However, this might not be the case for the velocity variable *normalized peak amplitude*, as the only study that examined this outcome had the largest sample size with a *n* = 118 for the INP group, but could not determine an effect [71]. Lacking of statistical power cannot explain limited and conflicting results for movement smoothness, as most studies had larger sample sizes (> 20 per group), and one study even pointed into the opposite direction, as the authors determined a decrease in the *number of velocity peaks* in NP, while for all other movement smoothness variables either an increase or no differences were reported (Table 10). Opposing results by just one study have also been found for *point deviation* in studies on movement accuracy [67]. Meisingset et al. interpreted a decreased *point deviation* as a “stiffening pattern” in INP [67] that, however has not been confirmed in a follow-up study by the same authors [38]. Another study by de Zoete et al. did not report opposing results but found no group differences for that same outcome variable [59].

Our systematic review is, for the most part, in line with recent reviews on further sensorimotor control variables, predominantly joint position sense, examined in NP versus healthy controls [10, 18, 23, 24]. While de Vries’ review focussed solely on joint position sense [18], others [10, 23, 24] reported also on further variables, some similar to our review, such as velocity [10], and movement accuracy [10, 23, 24]. Hesby et al. included ten studies on either *peak* or “average” *velocity*, and reported conflicting results too [10]. Some studies found lower velocity values for NP, while other studies did not [10]. Although the authors included studies on WAD, they did not provide separate results for INP and WAD, as we have done, if possible [10]. Within our review, we could determine a moderate level of evidence for a decreased *mean velocity* in WAD, provided by four studies [62, 69, 70, 80], of which only one had been included in Hesby et al’s review too [10]. Movement accuracy studies have been examined by all three reviews [10, 23, 24]. They included two [23], three [24], or four primary studies respectively [10], all of them have also been included in this review [64, 65, 67, 74], and led, together with additional four studies, to a strong level of evidence for an increased number of errors in INP and WAD, while performing a head aiming (tracking) task (Table 11). Furthermore, an additional review by Moghaddas et al. focused solely on kinematics during functional movements, [22]. That review finally included five primary studies [22] of which two, assessing time-domain related kinematics, have also been included in our review [73, 79]. However, we regarded one of those as performing a head aiming, instead of a functional task [73]. In summary, most sensorimotor control variables examined in aforementioned reviews, demonstrated only little discriminatory validity such as for joint position sense [10, 18, 24] or postural stability [24]. The current review adds to research on sensorimotor control in NP and its expression within kinematic quantities, and movement accuracy. Moreover, it gives more distinct reference to the kind of movement task and outcome variables to be examined within their superior kinematic quantity.

### Strength and limitations

A strength of this review is the well-documented and methodological approach to a field of study troubled by high heterogeneity and uncertainties. Furthermore, the literature search was intentionally broad to be as encompassing as possible. Therefore, this review includes acute and chronic NP. However, most included articles reported on NP with a duration of 6 weeks or longer, so generalization and applicability to an acute NP condition remains limited. Another consequence of the broad literature search strategy was to include NP with both idiopathic and whiplash associated onset.

If possible, findings for kinematics and movement accuracy were presented separately for patients with WAD and INP. However, not all studies specified on whiplash grades according to the Quebec task force classification [84]. For those studies that did not distinguish WAD and INP, results were summarized as unclassified NP, and added to INP comparisons, which could have biased results for these outcome variables and NP groups. However, and as has been discussed before, for some outcome variables this review could demonstrate larger sensorimotor control differences in patients with WAD when compared to controls.

The level of evidence for summary findings was defined by a slightly adapted version of a classification system presented by the method guidelines for systematic reviews in the Cochrane collaboration Back Review Group [56]. According to both, the original and the adapted system, the level of evidence would classify as ‘conflicting’ if studies had showed opposing results, independent of the number or quality of these studies. In general, this has led to a stricter interpretation of results, since only one contradicting study would lead to a conflicting level of evidence rating. This approach has been favoured due to the heterogeneity of the studies and to be cautious in generalising the findings from kinematics and movement accuracy in patients with NP and WAD across tasks.

There are some limitations to this review. Due to the lack of an appropriate and validated RoB tool for cross sectional case control studies, and since there is no reference standard for assessing head kinematics or movement accuracy, an existing RoB tool that has been used in reviews with similar topics to this one [22, 24] was adapted for the purpose of this review [53]. This adapted version has not been validated, which limits comparison to other reviews. Nevertheless, the interrater reliability between the two reviewers was high and a detailed description of the tailoring process, as provided in the appendices, ensures reproducibility of the quality assessment. Furthermore, no weighting according to sample and effect sizes was included for quality assessment, which would have increased the precision of the quality rating.

Another limitation is that only statistically significant group differences have been considered, without discussion of their clinical relevance. Additionally, no generalized cut off values were presented for the determination of abnormal head motion kinematics or movement accuracy values between groups. Owing to the heterogeneity of included studies, as mentioned before, this was not regarded possible. Therefore, the practical benefit to clinicians is limited at this stage of research.

Finally, one limitation derives from the nature or entity of the topic itself, as previously mentioned. The high variability between included studies for movement tasks and outcome variables, and further specifications for both, combined with differing measurement technologies used, makes it difficult to draw firm conclusions on head kinematics or movement accuracy in NP.

### Implications for future research and clinical practice

Future research should standardize the measurements for the assessment of head motion kinematics, which would establish a base for the replication of methods to validate previous results. Furthermore, to increase confidence in the evidence, the focus should be on improving the methodological quality of studies. Sampling must include a detailed description of the screening procedure and participation rate. The included population should not differ between the groups, except for the condition under study. Furthermore, an a priori sample size should be determined. Measurement procedures need to be described in detail and should include test results from reliability studies. Furthermore, interactions between movement characteristics, such as velocity, displacement and direction need to be reported. Data analysis should be performed with group blinding. Matching, or statistical stratification, for confounding factors should be implemented. Studies are needed that relate kinematic and movement accuracy outcome variables to patient reported outcome variables, such as pain or disability.

Clinicians should consider the movement task which might be used within their setting, along with specifications. This also depends on the availability of technology, which might not be given in all settings. In addition, the evidence from longitudinal studies on the responsiveness of some kinematic or movement accuracy measures and in relation to changes in pain and disability is still controversial [27, 35, 38].

## Conclusion

Sensorimotor control in NP in the way of kinematic and movement accuracy characteristics of head motion was examined in head aiming, functional or unconstrained movement tasks.

Specific outcome variables under investigation, describe characteristics of velocity and acceleration, temporal characteristics, movement smoothness, and movement accuracy. The methodological quality of included studies was moderate and confidence in the level of evidence for outcomes ranged from strong to conflicting.

The results from this review indicate that for some characteristics that describe sensorimotor control, patients with NP differ from healthy controls, as strong evidence has been found for patients with INP and WAD to deviate more often from a tracking path than controls, with further strong evidence showing, that patients with INP need more time to complete a movement task. Moderate evidence indicates that acceleration in general, and during unconstrained movement tasks in NP, and specifically reaction time, mean velocity, peak acceleration as well as point deviation and time on a target differ between patients with WAD and controls, while movement smoothness variables have not been found to differ between patients with NP and control participants, so far.

## Supplementary Information

**Additional file 1.** Search strategy used for MEDLINE database

**Additional file 2.** Adapted version of the Quality Assessment Tool for Observational Cohort and Cross-Sectional Studies

## Data Availability

All data are available at the ZHAW (Zurich University of Applied Sciences) on application to the corresponding author, Markus J. Ernst (markus.ernst@zhaw.ch).

## Abbreviations

NP: Unclassified neck pain
INP: Idiopathic neck pain
RoB: Risk of bias
WAD: Whiplash associated disorder
