# Correction: Head kinematics in patients with neck pain compared to asymptomatic controls: a systematic review

**DOI:** 10.1186/s12891-022-05395-6

**Published:** 2022-05-24

**Authors:** Esther Franov, Matthias Straub, Christoph M. Bauer, Markus J. Ernst

**Affiliations:** Zurich University of Applied Sciences, School of Health Professions, Institute of Physiotherapy, Katharina-Sulzer-Platz 9, 8400 Winterthur, Switzerland

**Correction to: BMC Musculoskelet Disord 23, 156 (2022)**

**https://doi.org/10.1186/s12891-022-05097-z**

Following publication of this [1] article, the authors report the following corrections to the abstract section (i,ii) and Table 4 (iii):

i) The authors would like to correct and precise their statement in the results section of the abstract regarding increased *movement* time and increased *number of errors* during head aiming tasks in neck pain patients when compared to control subjects: as indeed the *number of errors* was increased across all neck pain patients (both idiopathic and whiplash associated neck pain) with a strong level of evidence, the strong level of evidence for an increase in *movement time* was only found in idiopathic neck pain patients. Results in whiplash associated neck pain patients regarding *movement time* were inconsistent, as the authors described in detail in the result and conclusion section of the main text.
ii) The authors noted that an imprecision occurred in summarizing results in the abstract section leading to a potential misinterpretation of the results by the readers (”moderate evidence was found in traumatic neck pain for a decreased mean velocity, peak acceleration, and reaction time, and for point deviation and time on target during head aiming tasks”): indeed moderate evidence was found in whiplash associated neck pain patients for altered *mean velocity*, *peak acceleration*, *reaction time*, *point deviation* and *time on target* during head aiming tasks, but these alterations implied on one hand a decrease (*mean velocity*, *peak acceleration* and *time on target*), whereas on the other hand an increase (*reaction time* and *point deviation*), as correctly described in detail in the results and conclusion section of the main text.
iii) The authors would like to add a correction to Table 4 (Participants, demographics and clinical characteristics across included studies): the samples of Yang et al [2] and Zhou et al [3] are here wrongly stated as idiopathic neck pain patients (INP), but were in fact “not classified concerning their neck pain origin”, being either idiopathic or whiplash associated. In the analysis however, both studies have been correctly treated and described as studies comparing healthy controls with unclassified neck pain patients. Therefore, the review results and conclusions remain unchanged.

The original article [1] has been updated.
