# Supplementary material for: Head kinematics in patients with neck pain compared to asymptomatic controls: a systematic review
Source: BMC Musculoskelet Disord. 2022 Feb 16;23:156. doi: 10.1186/s12891-022-05097-z (PMC8848642; doi:10.1186/s12891-022-05097-z)
Supplement: Supplementary file 1 — Additional file 1. Search strategy used for MEDLINE database [file 12891_2022_5097_MOESM1_ESM.docx]

**Additional file 1: Search strategy**

| Search No. | Searches |
| --- | --- |
| 1 | exp Cervical Vertebrae/in, pp [Injuries, Physiopathology] |
| 2 | Head/pp [Physiopathology] |
| 3 | Neck/pp [Physiopathology] |
| 4 | Neck Pain/ |
| 5 | exp Neck Injuries/ |
| 6 | Neck Muscles/pp [Physiopathology] |
| 7 | Atlanto-Axial Joint/pp [Physiopathology] |
| 8 | Atlanto-Occipital Joint/pp [Physiopathology] |
| 9 | 1 or 2 or 3 or 4 or 5 or 6 or 7 or 8 |
| 10 | Movement/an, cl, ph [Analysis, Classification, Physiology] |
| 11 | Head Movements/ph [Physiology] |
| 12 | Physical Examination/mt [Methods] |
| 13 | Motor Skills/ph, pp [Physiology, Physiopathology] |
| 14 | Proprioception/ph [Physiology] |
| 15 | Kinesthesis/ |
| 16 | Exercise Movement Techniques/ |
| 17 | "Range of Motion, Articular"/ |
| 18 | Biomechanical Phenomena/ |
| 19 | 10 or 11 or 12 or 13 or 14 or 15 or 16 or 17 or 18 |
| 20 | "Reproducibility of Results"/ |
| 21 | "Sensitivity and Specificity"/ |
| 22 | Validation Studies/ |
| 23 | Validation Studies as Topic/ |
| 24 | Cross-Sectional Studies/ |
| 25 | Case-Control Studies/ |
| 26 | Diagnostic Errors/ |
| 27 | Observer Variation/ |
| 28 | 20 or 21 or 22 or 23 or 24 or 25 or 26 or 27 |
| 29 | 9 and 19 and 28 |
| 30 | limit 29 to ("all adult (19 plus years)" and english and (female or humans or male)) |
