# Supplementary material for: Head kinematics in patients with neck pain compared to asymptomatic controls: a systematic review
Source: BMC Musculoskelet Disord. 2022 Feb 16;23:156. doi: 10.1186/s12891-022-05097-z (PMC8848642; doi:10.1186/s12891-022-05097-z)
Supplement: Supplementary file 2 — Additional file 2. Adapted version of the Quality Assessment Tool for Observational Cohort and Cross-Sectional Studies [file 12891_2022_5097_MOESM2_ESM.docx]

**Additional file 2: Adapted form of the Quality Assessment Tool for Observational Cohort and Cross-Sectional Studies**

|  | **Original items** | **Specifications and adaptations exclusively for this review** | **Article section** |
| --- | --- | --- | --- |
| 1, | Was the research question or objective in this paper clearly stated? |  |  |
| 2. | Was the study population clearly specified and defined? | Demographic information, including description of age by range OR by mean and SD if clearly stated that only adults were included, description of recruitment location and timeframe | Selection |
| 3. | Was the participation rate of eligible persons at least 50%? | Number of persons fitting the eligibility criteria and the rate of those consenting to participate | Selection |
| 4.* | Were all the subjects selected or recruited from the same or similar populations (including the same time period)?  Were inclusion and exclusion criteria for being in the study prespecified and applied uniformly to all participants? | The two questions were divided into separate items to better represent differences between the studies  Beside the definition for cases and controls, further inclusion and exclusion criteria had to be exactly the same for both groups | Selection |
| 5. | Was a sample size justification, power description, or variance and effect estimates provided? | An a priori sample size calculation had to be provided | Selection |
| 6. | For the analyses in this paper, were the exposure(s) *^a^* of interest measured prior to the outcome(s) *^a^* being measured? | All included studies being cross sectional analyses, this item has been omitted | - |
| 7. | Was the timeframe sufficient so that one could reasonably expect to see an association between exposure and outcome if it existed? | All included studies being cross sectional analyses, this item has been omitted | - |
| 8. | For exposures that can vary in amount or level, did the study examine different levels of the exposure as related to the outcome (e.g., categories of exposure, or exposure measured as continuous variable)? | Correlation analysis between severity of neck patient’s impairment (pain or disability) and outcome  OR regression analysis with neck pain or intensity as covariate | Statistics |
| 9.* | Were the exposure measures (independent variables) clearly defined, valid, reliable, and implemented consistently across all study participants? | Were the cases (neck patients) clearly defined and differentiated from the controls?  Were the controls therefore described in detail in respect to neck pain status? | Method |
| 10. | Was the exposure(s) assessed more than once over time? | All included studies being cross sectional analyses, this item has been omitted | - |
| 11.* | Were the outcome measures (dependent variables) clearly defined, valid, reliable, and implemented consistently across all study participants? | The validity of the outcome measures previously being confirmed was not rated as criterion in this review, representing itself a part of the primary studies research questions in the sense of assessing discriminating validity  Detailed description of the outcome measurement method to be reproducible (posture, instruction, device, repetitions, patterns) and applied uniformly to cases and controls  Reliability of outcome assessed in the study itself or information about reliability cross referenced | Method |
| 12. | Were the outcome assessors blinded to the exposure status of participants? | Including Lab personnel and statisticians, who further process and analyse data. | Statistics |
| 13. | Was loss to follow-up after baseline 20% or less? | All included studies being cross sectional analyses, this item has been omitted | - |
| 14.* | Were key potential confounding variables measured and adjusted statistically for their impact on the relationship between exposure(s) and outcome(s)? | Were all measurements controlled for age and gender?  For the outcomes of mean and peak velocity/acceleration in tasks with the instruction to move as fast as possible, has been controlled for displacement or range of motion?  For the outcome of movement smoothness, has been controlled for velocity? | Statistics |
|  | **Additional items** | |  |
| 1.* | Exclusion criteria prespecified to control for comorbidity of diseases with potential influence on examined head movement characteristic? | - Vestibular disorders and dysfunction - Neurological or central nervous system conditions - Disorders of eye movements or (uncorrected) visual impairments (in studies where tasks required a visual attention on objects) | Selection |
| 2. | Appropriate descriptive statistics presented? | Description of participants characteristics  Description of exposure (if assessed with continuous variable, e.g. via NRS or NDI) and outcome variables with mean and dispersion (range or SD) | Statistics |
| 3.* | Measurements of exposure (neck pain or neck disability) were tested on the same day as the outcome across all study participants? | Was the stability of the variables compared guaranteed? | Method |

*^a^* Several items refer to “exposure” and “outcome”, as the tool was primarily designed for cohort studies. For this review’s purpose, exposure was defined as being a neck patient, outcome defined as the head movement characteristic variables.

*These items were considered to weight more heavily in the rating of methodological quality of included studies
